# Comparative Effectiveness of Piperacillin-Tazobactam or Fluoroquinolones Versus Third-Generation Cephalosporins in Spontaneous Bacterial Peritonitis: A Systematic Review and Meta-Analysis

**DOI:** 10.7759/cureus.99305

**Published:** 2025-12-15

**Authors:** Jie Siang See, Sin Yee Wong

**Affiliations:** 1 Acute Internal Medicine, Northwick Park Hospital, London, GBR; 2 Old Age Psychiatry, Cefn Coed Hospital, Swansea, GBR

**Keywords:** ascites, fluoroquinolones, piperacillin-tazobactam, spontaneous bacterial peritonitis (sbp), third generation cephalosporins

## Abstract

Spontaneous bacterial peritonitis (SBP) remains a significant cause of morbidity and mortality among patients with cirrhosis. Third-generation cephalosporins (TGCs) have long been considered standard empiric therapy in major guidelines such as the British Society of Gastroenterology (BSG), the European Association for the Study of the Liver (EASL), and the American Association for the Study of Liver Diseases (AASLD). However, rising resistance patterns have led to increased use of piperacillin-tazobactam within the National Health Service (NHS). We aimed to evaluate the current evidence on the use of piperacillin-tazobactam and fluoroquinolones (FQs) in SBP through a systematic review of randomised controlled trials (RCTs) and observational studies.

The study followed Preferred Reporting Items for Systematic Review and Meta-Analysis (PRISMA) 2020 guidelines. MEDLINE, EMBASE, and Cochrane Central Register of Controlled Trials (Cochrane CENTRAL) were searched from inception. The primary outcome was resolution of infection, with the secondary outcome being mortality (in-hospital or 30-day). Meta-analysis was performed where appropriate. Risk of bias was assessed using the Cochrane Risk of Bias 2 (RoB 2) and the Risk Of Bias In Non-randomized Studies of Interventions (ROBINS-I) tools, and evidence certainty was evaluated using the Grading of Recommendations, Assessment, Development and Evaluation (GRADE) framework.

Nine studies (n = 2034) met the inclusion criteria. There was no difference between FQs and TGCs in terms of resolution of infection (odds ratio (OR) 0.95 (0.67-1.34), P = 0.90, I^2^ = 0%) and mortality (OR 0.76 (0.46- 1.24), P = 0.27, I^2^ = 0). Resolution of infection data for piperacillin-tazobactam versus TGCs was narratively reviewed, showing non-inferiority in a cohort and higher microbiological coverage in a culture-positive SBP cohort. The certainty of the evidence was very low according to the GRADE framework. No study directly reported mortality for piperacillin-tazobactam in SBP. One included study examined purely nosocomial SBP and showed a significantly higher rate of infection resolution with meropenem plus daptomycin versus ceftazidime. In summary, in community-acquired SBP, FQs are comparable to TGCs in terms of resolution of infection and mortality. Piperacillin-tazobactam may provide non-inferior clinical efficacy and broader microbiological coverage in settings with a higher prevalence of resistant or nosocomial organisms. Wider-spectrum coverage should be considered for nosocomial infections or patients with severe disease, guided by local antibiotic resistance patterns. However, no study reported mortality outcomes specifically for piperacillin-tazobactam. The low certainty of evidence for piperacillin-tazobactam versus TGCs highlights the need for well-powered RCTs.

## Introduction and background

Spontaneous bacterial peritonitis (SBP) is a common complication in patients with cirrhosis and ascites, with a global prevalence of 17% and a pooled mortality rate of 30% [1]. It is commonly caused by Gram-negative enteric bacteria, such as *Escherichia coli*, followed by *Klebsiella pneumoniae*, *Staphylococcus aureus*, *Enterococcus faecalis*, and *Enterococcus faecium*, with less than 5% due to fungal infection. Up to one-third of infections are due to multidrug-resistant (MDR) bacteria, with regional differences [2]. Third-generation cephalosporins (TGCs), including cefotaxime, ceftazidime and ceftriaxone, have been the first-line treatment suggested by the major guidelines such as the British Society of Gastroenterology (BSG), the European Association for the Study of the Liver (EASL), and the American Association for the Study of Liver Diseases (AASLD) for the management of community-acquired SBP [3-5]. However, emerging antimicrobial resistance, especially in nosocomial or healthcare-associated SBP, has raised concern about their continued efficacy. The above guidelines acknowledge the variability in local resistance patterns, and the choice of antibiotic should be guided by local resistance patterns and protocols. In the National Health Service (NHS), piperacillin-tazobactam is frequently used as first-line empiric therapy despite limited comparative evidence. A 2014 audit in the United Kingdom highlighted that 45.5% of NHS centres in Northwest England used piperacillin-tazobactam as first-line antibiotics for SBP [6].

A Cochrane network meta-analysis in 2019 evaluated various antibiotics for SBP [7]. To date, no meta-analysis has directly compared piperacillin-tazobactam or fluoroquinolones (FQs) with TGCs in the treatment of SBP. Given the widespread NHS use of piperacillin-tazobactam, alongside the evolving resistance landscape and the risk of *Clostridioides difficile* infection, a focused evaluation of its efficacy is needed. We aimed to evaluate the current evidence on the use of piperacillin-tazobactam and FQs in SBP compared to TGCs through a systematic review of randomised controlled trials (RCTs) and observational studies.

## Review

Methods

Protocol and Reporting Standards

This review was conducted according to the Preferred Reporting Items for Systematic Review and Meta-Analysis (PRISMA) statement 2020 [8]. The protocol was registered in PROSPERO (CRD420251162784).

Research Question and Population, Intervention, Comparison, and Outcome (PICO) Framework

The research questions were formulated using a PICO framework. The population (P) comprised adults (>18 years) with cirrhosis and SBP, defined by an ascitic fluid polymorphonuclear leukocyte (PMN) count > 250 cells/mm^3^, culture positive or negative, excluding paediatric, secondary, postoperative, or peritoneal dialysis-related peritonitis. The intervention (I) included piperacillin-tazobactam used empirically or as directed therapy for SBP, or FQs, such as ciprofloxacin and ofloxacin, used empirically or as directed therapy for SBP. The comparator (C) was TGCs, including cefotaxime, ceftriaxone, or ceftazidime, as per guideline-recommended standard therapy. The primary outcome (O) was resolution of infection (clinical resolution or PMN < 250 cells/mm^3^), while the secondary outcome was mortality (in-hospital or at 30-day). Although we aimed to stratify outcomes by culture status, most studies did not report whether SBP was culture-confirmed or presumed, which prevented us from performing this subgroup analysis.

Search Strategy

We used MEDLINE (Ovid), EMBASE (Ovid), and Cochrane Central Register of Controlled Trials (CENTRAL) to identify relevant studies on SBP and antibiotic comparisons. The search strategy is provided in Table 1. Publications not written in the English language were excluded. We included RCTs and comparative observational studies, including prospective or retrospective cohorts and case-control studies. Single-arm, case series, case reports, reviews, editorials, and animal or in vitro studies were excluded. The references of included papers and relevant reviews were hand-searched for additional papers. In addition to the above database searches, reference lists and publisher websites were screened to identify potentially relevant but non-indexed studies. Authors of papers identified in the search strategy were contacted for additional data as required.

**Table 1:** Search strategy for MEDLINE, EMBASE and Cochrane Central database The .mp. field tag searches multiple fields (title, abstract, MeSH/Emtree headings, keywords). Steps 1-4 identify relevant studies on spontaneous bacterial peritonitis and antibiotic comparisons. Steps 5-6 restrict to human studies and studies published in English only. Step 7 limits to clinical or observational comparative studies. MeSH: medical subject headings

Step	Search String
1	("spontaneous bacterial peritonitis" OR "spontaneous peritonitis" OR SBP OR "bacterial peritonitis" OR "infection of ascitic fluid").mp.
2	(cirrhosis OR cirrhotic OR ascites OR "liver disease").mp.
3	(antibiotic* OR antibacterial* OR antimicrobial* OR "empirical therapy" OR "empiric therapy" OR cefotaxime OR ceftriaxone OR ceftazidime OR "third generation cephalosporin*" OR tazocin OR "piperacillin tazobactam" OR fluoroquinolone* OR quinolone* OR ciprofloxacin OR ofloxacin OR levofloxacin).mp.
4	1 AND 2 AND 3
5	Limit 4 to humans
6	Limit 5 to English
7	Limit 6 to (clinical study OR comparative study OR cohort study OR case-control study)
8	Remove duplicates
9	Screen titles and abstracts for relevance according to the inclusion criteria
10	Retrieve full texts of eligible studies for detailed assessment
11	Hand-search reference lists and relevant reviews for additional studies

Eligibility Criteria

The study inclusion criteria were adults aged 18 years or older with cirrhosis and SBP, as defined by ascitic fluid PMN count of more than 250 cells/mm^3^, with culture results positive or negative. Exclusion criteria were paediatric populations, secondary peritonitis, post-operative peritonitis, and peritoneal dialysis-related infections. The primary outcome was resolution of infection, as defined as clinical resolution or PMN less than 250/mm^3^. The secondary outcome was mortality (in-hospital or 30-day).

Study Selection

Titles and abstracts were independently screened against eligibility criteria by both authors. Full texts of qualifying papers were downloaded and reviewed. Disagreements were resolved at all stages.

Risk of Bias Assessment

Risk of bias was evaluated using the Cochrane Risk of Bias 2 (RoB 2) for RCTs and the Risk Of Bias In Non-randomized Studies of Interventions (ROBINS-I) for observational studies.

Data Synthesis

Data extraction was performed independently by two reviewers. Data analysis was performed using Review Manager (RevMan) Version 7.2.0 (The Cochrane Collaboration, London, England, UK). A random-effects meta-analysis was performed where appropriate, using odds ratios (ORs) with 95% confidence intervals (CIs). The I^2^ test was used to assess heterogeneity, with values of 0.25, 0.50, and 0.75 indicating low, moderate, and high heterogeneity, respectively. Certainty of evidence was assessed using the Grading of Recommendations, Assessment, Development, and Evaluation (GRADE) framework [9].

Results

Study Selection Process

**Figure 1:**
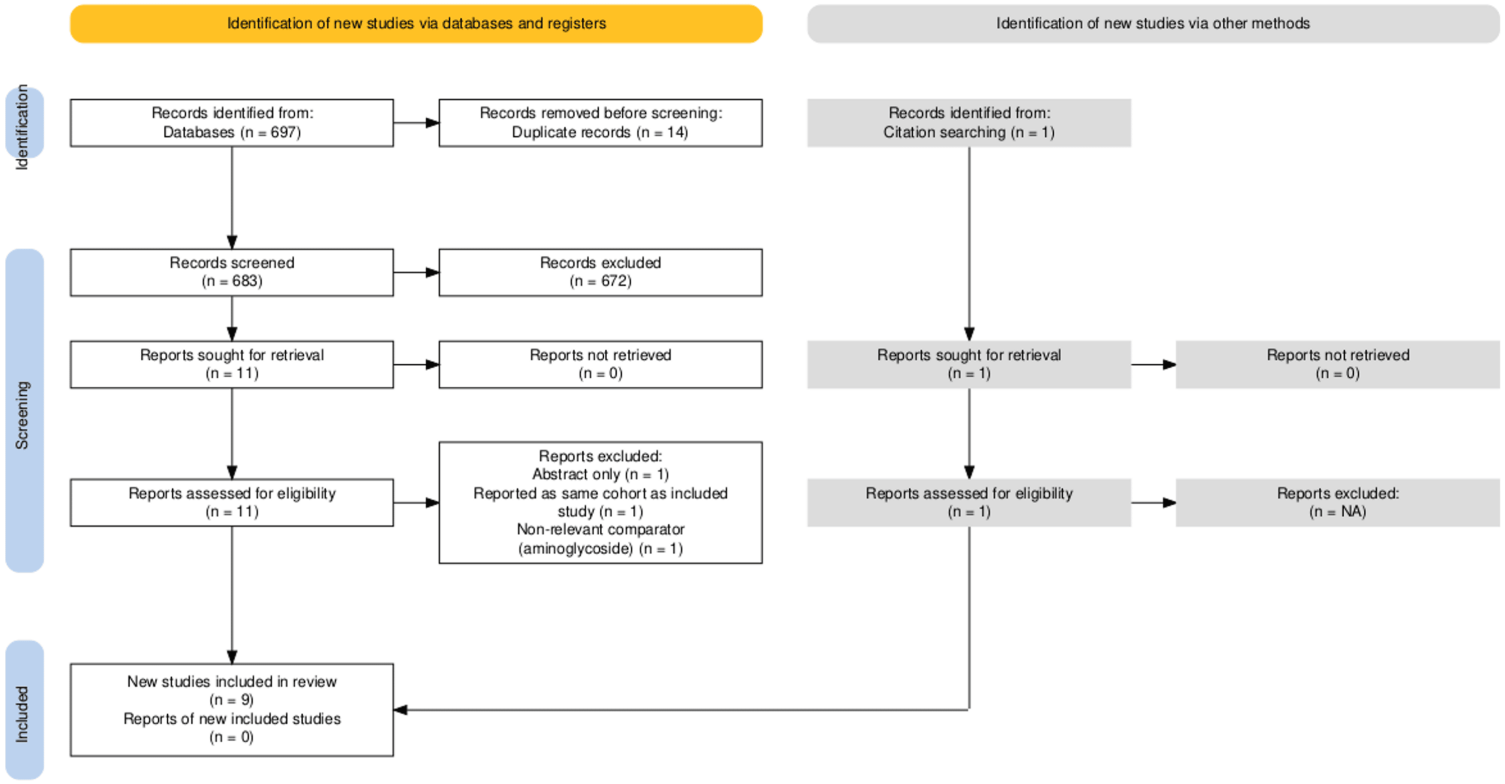
Preferred Reporting Items for Systematic Reviews and Meta-Analyses (PRISMA) 2020 flow diagram of study selection

The search of the literature and study selection is outlined in the PRISMA flow diagram, generated using a Shiny app (Stockholm Environment Institute, Stockholm, Sweden) for producing PRISMA 2020-compliant flow diagrams (see Figure 1) [10]. The titles and the abstracts of 697 identified records were screened. Fourteen duplicates and 672 non-relevant studies were removed. In addition to the database searches, reference lists and relevant journal websites were also reviewed to ensure that studies that were not indexed were not missed. This supplementary search identified one additional study, which was assessed and included after confirming that it met our eligibility criteria. Full texts of 12 studies were retrieved. Three studies were excluded after review of the full text. One reported only an abstract, one reported on the same cohort as the included study, and one with a non-relevant comparator (aminoglycoside). Nine studies were included.

Characteristics of the Included Studies

The summary of included studies is outlined in Table 2. The total number of patients included in the data analysis was 2034 from nine studies, with sample sizes ranging from 30 to 865 participants. Of the nine studies [11-19], four were RCTs [11,13,16,18], two were prospective studies but not randomised [14,17], and three were retrospective observational studies [12,15,19]. Seven studies were purely or predominantly community SBP, with community SBP ranging from 65% to 82% [11,12,14,16-19]. Novovic et al.’s cohort consisted of a mixed population of community-acquired and nosocomial SBP, with a distribution of 54% and 46%, respectively [15]. Piano et al. exclusively examined nosocomial infection [13]. All studies identified SBP patients based on clinical presentation and analysis of ascitic fluid cell counts and culture, except one, which identified episodes of SBP using culture-positive ascitic fluid samples from the laboratory [14]. Seven studies covered resolution of infection and mortality [11-13,15-18], with two studies only reporting resolution of infection [14,19]. For the interest of this meta-analysis, when multi-arm studies included overlapping antibiotic regimens sharing a control group, only the most clinically relevant or commonly used antibiotic comparison was included to avoid double-counting [12,15]. There were no commercially sponsored studies, and any conflicts of interest were noted from screening of the full text.

**Table 2:** Characteristics of the included studies RCT: randomised controlled trial; TGCs: third-generation cephalosporins (cefotaxime, ceftazidime and ceftriaxone); SBP: spontaneous bacterial peritonitis; FQ: fluoroquinolone

Author	Year	Country	Study Design	Sample Size (n)	Population (Community or Nosocomial)	Antibiotics	Outcomes Measured for Meta-Analysis
Yim et al. [11]	2023	South Korea	Multicentre RCT	261	Predominantly community: 213 (82%) community and 48 (18%) nosocomial	TGCs, ciprofloxacin	Resolution of infection, 30-day mortality
Kim et al. [12]	2021	Korea	Multicentre retrospective study	865	Predominantly community: 664 (77%) and 201 (23%) nosocomial SBP	TGCs, carbapenems, others (piperacillin-tazobactam, FQ, cefepime)	In hospital mortality
Piano et al. [13]	2016	Italy	Multicentre RCT	32	Nosocomial SBP	Meropenem + daptomycin, ceftazidime	Resolution of infection, 90-day transplant-free mortality
Ahmed et al. [14].	2014	Pakistan	Singe centre prospective study	240	Community-acquired SBP	Ciprofloxacin, ceftriaxone	Resolution of infection
Novovic et al [15]	2012	Denmark	Multicentre retrospective study of culture-positive SBP	140 (187 ascitic isolates)	Mixed: 76 (54%) community-acquired and 64 (46%) nosocomial SBP	TGCs, piperacillin-tazobactam, meropenem	30-day mortality, antibiotic susceptibility
Angeli et al. [16]	2006	Italy	Multicentre RCT	116	Predominantly 75 (65%) community and 41 (35%) hospital-acquired SBP	Ciprofloxacin, ceftazidime	Resolution of infection, In-hospital mortality
Taşkıran et al. [17]	2004	Turkey	Single-centre prospective study	30	Community-acquired SBP	Ofloxacin, cefotaxime	Resolution of infection, In-hospital mortality
Navasa et al. [18]	1996	Spain	Multicentre RCT	132	Predominantly community: 88 (71%) and 35 (29%) nosocomial	Ofloxacin, cefotaxime	Resolution of infection, in in-hospital mortality
Hussain et al. [19]	2025	Pakistan	Single-centre retrospective study	218	Community-acquired SBP	Piperacillin-tazobactam, ceftriaxone	Resolution of infection

Comparing FQs With TGCs

**Figure 2:**
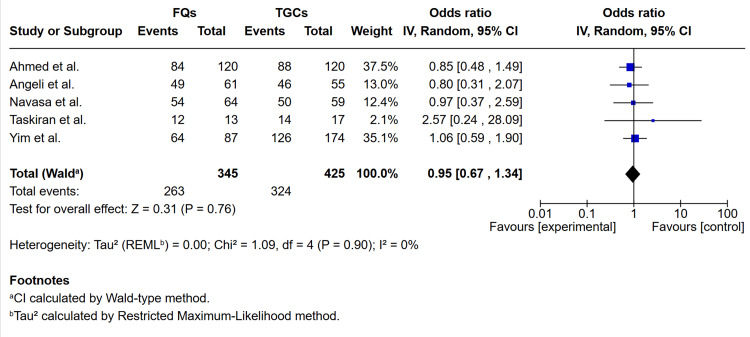
Forest plot comparing FQs and TGCs for resolution of infection in spontaneous bacterial peritonitis Studies included: Angeli et al. [16], Ahmed et al. [14], Navasa et al. [18], Taskiran et al. [17], Yim et al. [11]. FQs: fluoroquinolones (ciprofloxacin or ofloxacin); TGCs: third-generation cephalosporins (cefotaxime, ceftazidime or ceftriaxone); CI: confidence interval

Pooled data from five studies [11,14,16-18] comparing FQs with TGCs showed no significant difference in resolution of infection with OR 0.95 (0.67-1.34), P = 0.90, I^2^ = 0% (see Figure 2).

**Figure 3:**
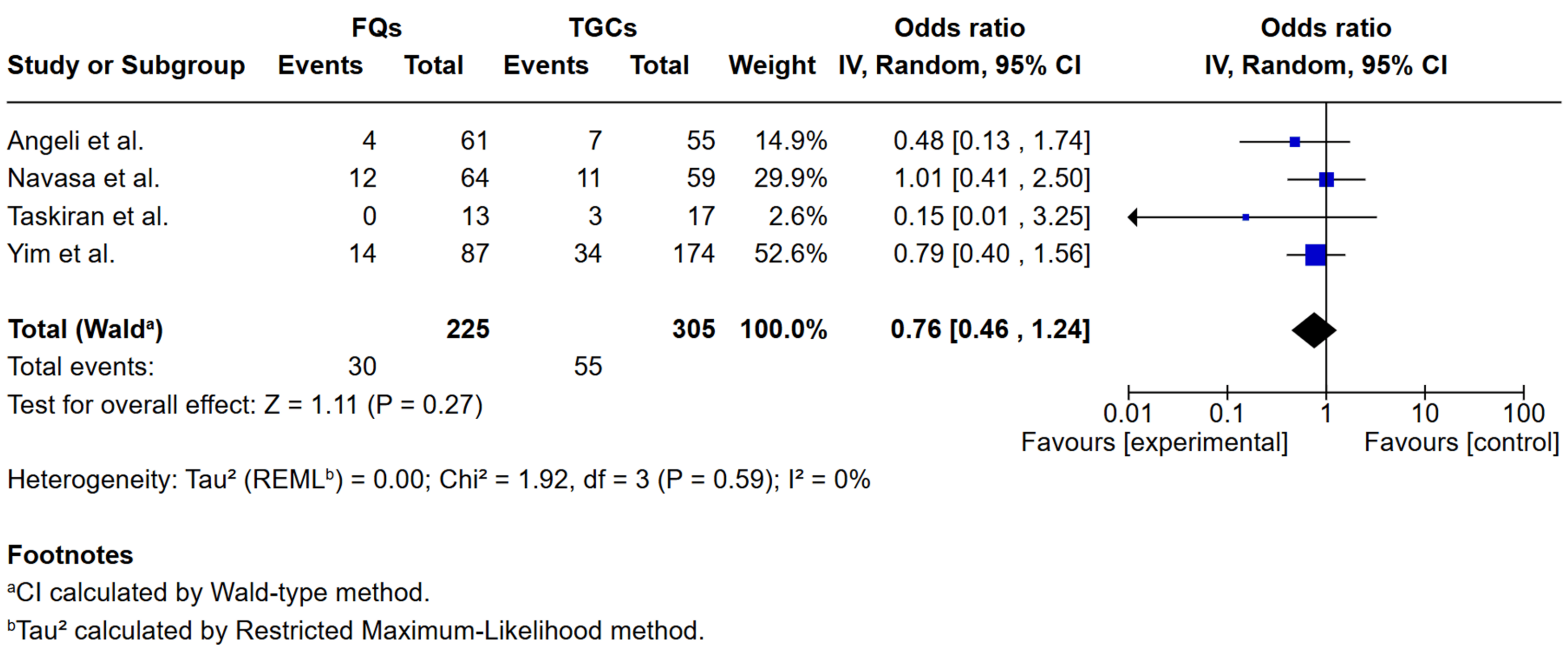
Forest plot comparing FQs versus TGCs for mortality in spontaneous bacterial peritonitis Studies included: Angeli et al. [16], Navasa et al. [18], Taskiran et al. [17], Yim et al. [13]. FQs: fluoroquinolones (ciprofloxacin or ofloxacin); TGCs: third-generation cephalosporins (cefotaxime, ceftazidime or ceftriaxone); CI: confidence interval

Pooled data from four studies [11,16-18] comparing FQs with TGCs showed no significant difference in terms of mortality, with OR 0.76 (0.46- 1.24), P = 0.27, I^2^ = 0% (see Figure 3).

Comparing Piperacillin-Tazobactam With TGCs

Hussain et al. [19] and Novovic et al. [15] compared TGCs with piperacillin-tazobactam. Hussain et al. reported clinical resolution as the primary outcome, whereas Novovic et al. focused on microbiological coverage, assessing whether the initial antibiotic regimen covered the causative organism. As the outcomes were not directly comparable, a formal meta-analysis was not performed. Instead, the results of these studies were narratively reviewed. Hussain et al. reported higher clinical resolution with piperacillin-tazobactam compared to ceftriaxone (75.2% vs. 62.4%), but the difference was not clinically significant (n = 218, P = 0.0574). Novovic et al. demonstrated higher microbiological coverage with piperacillin-tazobactam in their culture-positive cohort (73% susceptible to piperacillin-tazobactam vs. 57% susceptible to TGCs).

No study directly reported mortality for piperacillin-tazobactam in SBP. The only related evidence (Novovic 2012) evaluated mortality of covered (35%) versus non-covered (55%) culture-positive SBP, with P = 0.017 (Log-rank). We know from the above that there was higher microbiological coverage with piperacillin-tazobactam (73% susceptible to piperacillin-tazobactam vs. 57% susceptible to TGCs), but no direct data on piperacillin-tazobactam was available. Kim et al. reported mortality rates for TGCs, carbapenems, and piperacillin-tazobactam. However, the exact mortality data for the piperacillin-tazobactam cohort were not reported, as it was grouped as “others” with no specific mortality data provided [12].

Nosocomial SBP

Data for nosocomial SBP were descriptive only. The single available study (Piano et al.) compared meropenem-daptomycin with ceftazidime and found improved coverage in the meropenem-daptomycin cohort, 13/16 (86.7%), and in 4/16 (25%) in the ceftazidime cohort, P < 0.001. Ninety-day transplant-free survival was higher in the meropenem-daptomycin cohort (79.4% vs. 68.8%), but the difference was not statistically significant (P = not significant) [13]. Ineffective response to first-line treatment, acute kidney injury, and baseline mean arterial pressure were found to be independent predictors of 90-day transplant-free survival after multivariate analysis [12].

Risk of Bias Assessment

Risk of bias, assessed using RoB 2 for randomised trials and ROBINS-I for observational studies, is summarised (see Tables 3-4). The overall risk of bias among the included RCTs ranged from some concerns to high risk, mainly due to open-label designs, unclear allocation concealment, and occasional use of subjective clinical outcomes [11,13,14,16,18]. The observational studies demonstrated substantially greater bias, with serious to critical risk driven by confounding by indication, clinician-directed antibiotic choice, and selection of more severe or culture-positive cases [12,15,19].

**Table 3:** Risk of bias assessment of included randomized controlled trials using the Cochrane Risk of Bias 2 (RoB 2) tool Risk of bias was assessed as low risk, some concerns or high risk.

Study	Randomisation Process	Deviations From Intended Interventions	Missing Outcome Data	Measurement of Outcome	Selection of Reported Result
Yim et al. [11]	Some concerns	Some concerns	Low risk	Low risk	Low risk
Piano et al. [13]	Some concerns	Some concerns	Low risk	Low risk	Low risk
Ahmed et al. [14]	High risk	High risk	Some concerns	High risk	Some concerns
Angeli et al. [16]	Some concerns	Some concerns	Low risk	Low risk	Low risk
Navasa et al. [18]	Low risk	Some concerns	Low risk	Low risk	Low risk

**Table 4:** Risk of bias assessment of included observational studies using the Risk Of Bias In Non-randomized Studies of Interventions (ROBINS-I) tool Risk of bias was assessed as low, moderate, serious or critical risk.

Study	Confounding	Selection of Participants	Classification of Interventions	Deviations From Intended Interventions	Missing Data	Measurement of Outcomes	Selection of the Reported Result
Kim et al. [12]	Serious risk	Moderate risk	Low risk	Low risk	Low risk	Low risk	Low risk
Novovic et al. [15]	Serious risk	Serious risk	Low risk	Low risk	Low risk	Low risk	Low risk
Taskiran et al. [17]	Serious risk	Moderate risk	Low risk	Moderate risk	Low risk	Moderate risk	Moderate risk

Summary of Certainty of Evidence Using the GRADE Framework

Certainty of evidence was summarised and assessed using the GRADE framework (see Table 5) [9]. The certainty of evidence for resolution of infection in SBP when comparing FQs versus TGCs was moderate, with no serious risk of bias, inconsistency, indirectness, or imprecision, and no suspected publication bias [11,14,16-18]. For piperacillin-tazobactam versus TGCs, the evidence was of very low certainty due to risk of bias (non-randomisation, observational, and retrospective design) and non-comparable outcomes across the two studies [15,19].

**Table 5:** Summary of the certainty of evidence for all included studies assessed using the Grading of Recommendations, Assessment, Development and Evaluation (GRADE) framework The confidence in evidence is on a continuum from high certainty, moderate certainty, low certainty and very low certainty. Studies included: Yim et al. [11], Kim et al. [12], Piano et al. [13], Ahmed et al. [14], Novovic et al. [15], Angeli et al. [16], Hussain et al. [17], Navasa et al. [18]. FQs: fluoroquinolones (ciprofloxacin or ofloxacin); TGCs: third-generation cephalosporins (cefotaxime, ceftazidime or ceftriaxone); RCT: randomised controlled trial; OR: odds ratio; CI: confidence interval

Outcome	No. of Studies	Participants	Relative Effect (95% CI)	Certainty of Evidence Using the GRADE Framework
Resolution of infection (FQs vs. TGCs)	Five RCTs [11,14,16-18]	770	OR 0.95 (0.67-1.34), P = 0.90, I^2^ = 0%	Moderate
Resolution of infection (piperacillin-tazobactam vs. TGCs)	One retrospective observational study and one retrospective study of culture-positive SBP [15,19]	415	N/A due to non-comparable outcomes	Very low
Mortality- in-hospital or 30-day (FQs vs. TGCs)	Four RCTs [11,16-18]	530	OR 0.76 (0.46-1.24), P = 0.27, I^2^ = 0%	Moderate

Overall certainty remains moderate for the mortality of SBP when comparing FQs versus TGCs with no serious inconsistency or indirectness [11,16-18]. As no study directly measured mortality related to piperacillin-tazobactam, grading of the evidence was not possible.

Discussion

In our meta-analysis, we found that mortality and clinical resolution rates were similar between FQs and TGCs in community-acquired SBP. This continues to support its use as an alternative to TGCs as first-line treatment of community-acquired SBP, although its use should be avoided in patients taking FQs for SBP prophylaxis and in regions where antibiotic resistance to SBP is high [4].

Findings comparing the resolution of infection of piperacillin-tazobactam and TGCs were narratively reviewed. One study (Hussain et al.) described a higher rate of resolution of SBP with piperacillin-tazobactam compared to ceftriaxone, although it was statistically insignificant [19]. One study (Novovic et al.) identified SBP cases through culture-positive microbiology records, which may over-represent SBP with higher clinical severity [15]. However, clinical outcomes were evaluated according to standardised response criteria. The cohort was mixed (54% community-acquired and 46% nosocomial SBP), reflecting real-world practice in which empiric regimens are often applied across both settings. The observed mortality advantage or equivalent efficacy supports the pragmatic NHS trend towards piperacillin-tazobactam use. Our findings suggest that piperacillin-tazobactam may be advantageous in settings where resistant or nosocomial organisms are more prevalent. However, due to low certainty of evidence, no definite recommendations can be provided based on our findings.

Only one study (Piano et al.) included patients with purely nosocomial SBP. It showed that broader coverage of meropenem-daptomycin had a significantly higher rate of resolution than the ceftazidime cohort, resulting in early termination after the inclusion of only half of the provided sample size [13]. Kim et al. reported improved outcomes with carbapenems compared with TGCs in critically ill SBP patients with higher Chronic Liver Failure-Sequential Organ Failure Assessment (CLIF-SOFA) scores, with no overall mortality benefit in the general SBP population [12]. These findings align with the increasing prevalence of extended-spectrum beta-lactamase-producing *Enterobacteriaceae* in cirrhotic patients with prior antibiotic exposure or prolonged hospitalisation. This is supported by the AASLD guideline, which recommends piperacillin-tazobactam and daptomycin (if known vancomycin-resistant *Enterococcus* (VRE) in the past or evidence of gastrointestinal (GI) colonisation) or meropenem (if known to harbour MDR Gram-negative organisms) for nosocomial SBP.

A strength of this review is that it represented a comprehensive up-to-date analysis of studies investigating the outcomes of piperacillin-tazobactam or FQs versus TGCs in SBP. An exhaustive literature search was conducted, including both randomised and observational studies conducted over almost three decades, enabling us to capture shifts in antimicrobial practice and resistance patterns over time. Due to the limited available studies, meta-analysis was not possible when comparing piperacillin-tazobactam to TGCs, and the findings were narratively reviewed. Our meta-analysis provided a narrative synthesis of important outcomes across these studies and should be helpful to inform future research.

Limitations of this study included studies that varied in design, patient characteristics, and definitions of treatment success, which may have contributed to inconsistency in the pooled results, especially in the piperacillin-tazobactam cohort, where the certainty of evidence was low. In addition, confounding factors could not be controlled at the review level, particularly within the observational cohorts. Reporting of culture status was another important limitation. Many studies did not indicate whether SBP episodes were culture-positive or culture-negative, which meant we were unable to analyse outcomes according to microbiological confirmation. In addition, our search was restricted to English-language publications, which may introduce language and publication bias by excluding potentially relevant studies published in other languages, and may limit the completeness and generalisability of our findings.

Therefore, there is a clear need for well-powered multicentre RCTs comparing piperacillin-tazobactam and TGCs, with analyses stratified by clinical setting (community-acquired vs. nosocomial), disease severity, resistance risk, and adequate follow-up period to guide future updates to BSG, EASL, and AASLD recommendations.

## Conclusions

In community-acquired SBP, FQs are comparable to TGCs in terms of resolution of infection and mortality. Piperacillin-tazobactam may provide non-inferior clinical efficacy and broader microbiological coverage compared with TGCs, particularly in settings with a higher prevalence of resistant or nosocomial organisms. However, as no study directly reported mortality outcomes specifically for piperacillin-tazobactam, no conclusions can be drawn regarding mortality benefit. Broader-spectrum coverage should be considered for nosocomial or severely ill patients, guided by local antibiotic resistance patterns. While current evidence remains low certainty for the use of piperacillin-tazobactam empirically in community SBP, these results support its pragmatic use within the NHS settings. Future multicentre RCTs stratified by clinical setting (community-acquired vs. nosocomial), disease severity, and resistance risk, with adequate follow-up, are warranted for guideline reassessment.
